# MAP Kinase-Mediated c*-fos* Regulation Relies on a Histone Acetylation Relay Switch

**DOI:** 10.1016/j.molcel.2008.01.019

**Published:** 2008-03-28

**Authors:** Amanda O'Donnell, Shen-Hsi Yang, Andrew D. Sharrocks

**Affiliations:** 1 Faculty of Life Sciences, University of Manchester, Michael Smith Building, Oxford Road, Manchester M13 9PT, UK

**Keywords:** DNA, SIGNALING

## Abstract

Gene activation is often associated with high levels of histone acetylation. Enhanced acetylation levels can promote the recruitment of further chromatin modifying complexes or the basal transcription machinery. Here, we have studied MAP kinase-mediated upregulation of c*-fos* and uncover a role for histone acetylation in promoting the recruitment of a second transcription factor, NFI. MAP kinase signaling to Elk-1 enhances the net histone acetylase activity associated with the c*-fos* promoter, which leads to changes in the acetylation state and structure of a promoter-proximal nucleosome, which allows NFI binding. Binding of NFI provides a permissive state for the recruitment of basal machinery and subsequent promoter activation. Our results provide insights into how MAP kinase signaling promotes inducible gene expression; phosphorylation of recipient transcription factors (primary effectors) triggers a HAT relay switch, which facilitates the recruitment of additional transcription factors (secondary effectors) through alteration of the local nucleosomal structure.

## Introduction

The modification and remodeling of chromatin structure plays a pivotal role in the control of eukaryotic gene regulation (reviewed in Berger, 2002; Workman, 2006). Numerous histone modifications have been identified; one of which, acetylation, has been correlatively linked with gene activation. However, the molecular mechanisms through which enhanced histone acetylation levels cause target gene activation are largely unknown. To date, studies have focused on the role of acetylation in promoting the recruitment of additional regulatory proteins to chromatin through direct recognition of the acetylated lysine residues through motifs such as bromodomains (reviewed in Marmorstein and Berger, 2001). Other studies have demonstrated enhanced recruitment and activation of the basal machinery as a consequence of increased histone acetylation levels (Guermah et al., 2006). However, at the majority of promoters, it is still not clear whether histone acetylation plays additional roles in the transcriptional activation process.

MAP kinase signaling directly regulates the activities of numerous transcription factors through direct phosphorylation (reviewed in Yang et al., 2003a). One such transcription factor is the ETS-domain protein Elk-1. MAP kinase-mediated phosphorylation of Elk-1 triggers a number of molecular events that culminate in its activation. The allosteric activation of p300 and recruitment of mediator through the Med23 (Sur2) subunit are two pivotal events (Stevens et al., 2002; Li et al., 2003). In addition, signaling through the ERK MAP kinase pathway causes derepression of Elk-1 through reversing its sumoylation and hence promoting the dissociation of HDAC-2 (Yang et al., 2003b). Thus, one key process affected by MAPK signaling is the net output from the histone deacetylases (HDACs) and histone acetyltransferases (HATs) associated with Elk-1, leading to localized enhancement of acetylation levels at target promoters. One such well-studied target gene is c*-fos*, where histone acetylation levels within the promoter are usually in a dynamic equilibrium, permitting maintenance at a basal level, which is shifted toward high histone acetylation levels upon target gene activation (Clayton et al., 2000; reviewed in Clayton et al., 2006).

To better understand the role of histone acetylation in activation of the c*-fos* promoter, we studied the recruitment of factors to this promoter after MAP kinase pathway activation. Unexpectedly, we found that the transcription factor NFI is inducibly recruited to this promoter and is required for inducible c*-fos* activation. This induction is promoted by histone acetylation and requires the presence of Elk-1 and its associated HAT, p300. Thus, we have uncovered a novel mechanism by which MAP kinase signaling generates a transcriptional response through directly phosphorylating an acceptor transcription factor (here Elk-1), which leads to the indirect recruitment of a second transcription factor (NFI) via the triggering of a histone acetylation relay switch.

## Results

### MAP Kinase Signaling Induces Recruitment of NFI to the c*-fos* Promoter

**Figure 1:**
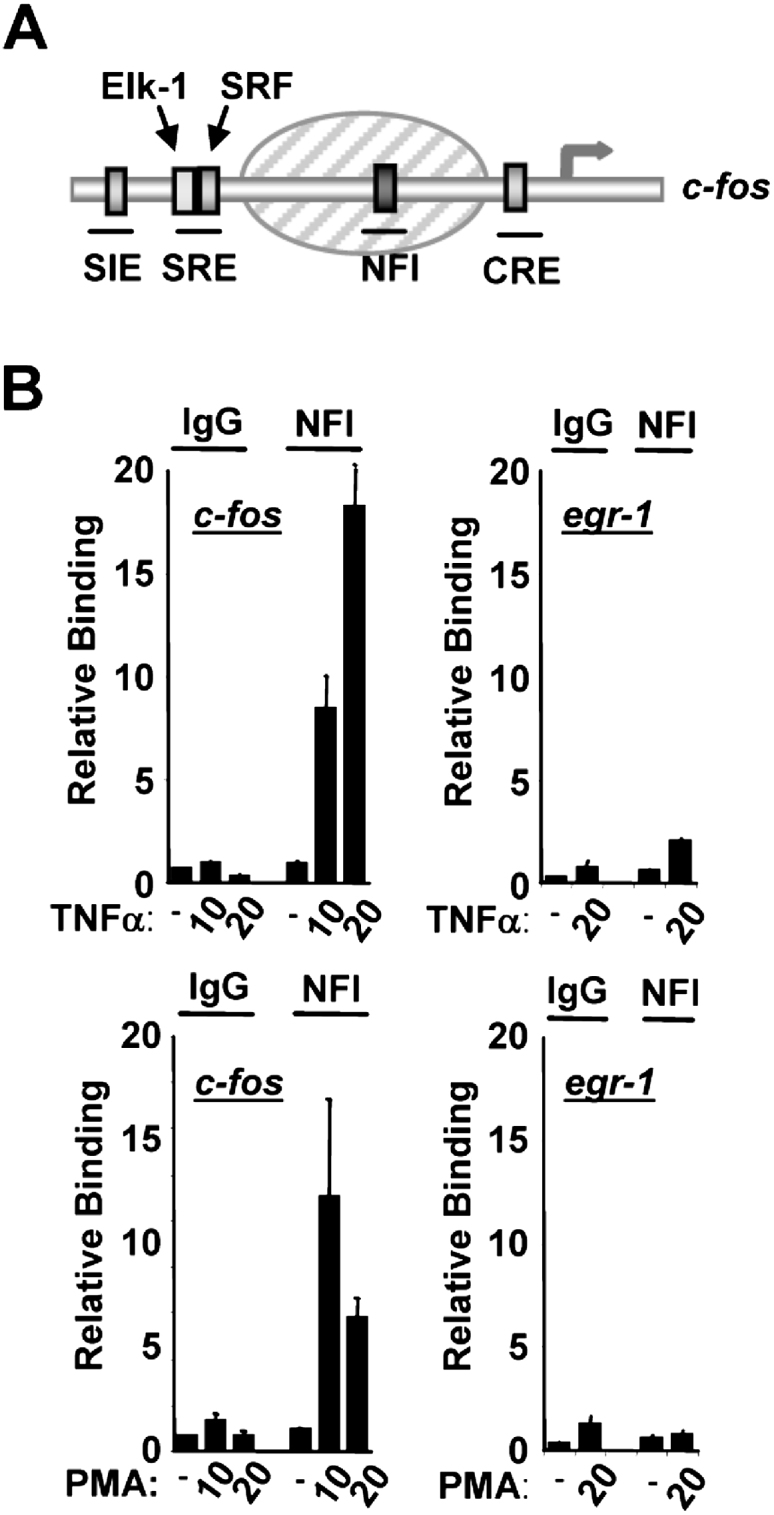
NFI Is Specifically Recruited to the c*-fos* Promoter in Response to MAP Kinase Activation (A) Schematic diagram of the c-*fos* promoter showing the locations of *cis*-regulatory elements. The shaded oval indicates the siting of a positioned nucleosome. (B) ChIP of NFI bound to the c-*fos* promoter. HeLa cells were starved in serum-free DMEM (−) or stimulated with either TNFα or PMA for the indicated times (min). Sonicated chromatin was immunoprecipitated with either an anti-NFI antibody or nonspecific IgG. PCR analysis of eluted DNA was performed with oligonucleotides specific for the c-*fos* promoter (left panels) or *egr-1* promoter (right panels). Data are presented as means ± SEM (n ≥ 4) and are the average of at least two independent experiments performed in duplicate.

The c*-fos* promoter contains a positioned nucleosome located between the Elk-1 binding site within the serum response element (SRE) and the promoter (Figure 1A; Herrera et al., 1997). Histones within this nucleosome therefore represent likely recipients of the enhanced acetylation output from Elk-1 after its phosphorylation in response to MAP kinase pathway signaling. This region of the c*-fos* promoter also contains a potential binding site for the transcription factor NFI, which has previously been shown to be important for basal promoter activity (Figure 1A; Lucibello et al., 1991; Runkel et al., 1991).

We first used chromatin immunoprecipitation (ChIP) assays to ask whether binding of NFI could be detected at the c*-fos* promoter in vivo. PMA was used to specifically activate the ERK MAP kinase pathway and TNFα to activate the p38 and JNK MAP kinase pathways (Figure S1C available online). In the absence of MAP kinase signaling, little NFI binding to the c*-fos* promoter could be detected (Figure 1B). However, rapid induction of NFI binding was observed after either TNFα or PMA treatment. In contrast, NFI binding was not enhanced by either stimulus at another Elk-1 target gene, *egr-1* (Figure 1B). Importantly, recruitment of NFI was dependent on MAP kinase signaling, as pharmacological inhibition of the ERK and JNK MAP kinase pathways blocked the inducible recruitment of NFI after PMA and TNFα stimulation, respectively (Figure S1).

Thus, MAP kinase signaling leads to the enhanced recruitment of NFI to the c*-fos* promoter in a region that is occupied by a nucleosome.

### Histone Acetylation Promotes NFI Recruitment

**Figure 2:**
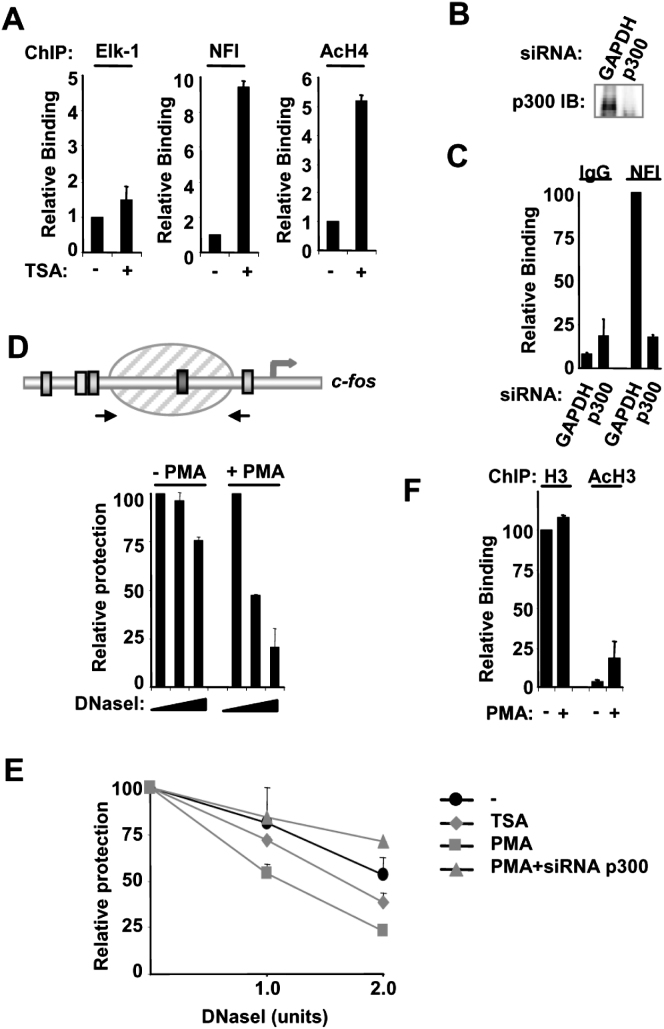
p300-Mediated Histone Acetylation Promotes Recruitment of NFI to the c*-fos* Promoter (A) ChIP showing the association of NFI, Elk-1, and acetylated histone H4 with the c*-*fos promoter upon TSA treatment. HeLa cells were serum starved (−) or starved and treated with TSA for 30 min (+) where indicated. (B) Immunoblot showing the knockdown of p300 protein expression in HeLa cells 48 hr after transfecting with siRNAs targeting p300. (C) ChIP of the c*-fos* promoter with either an antibody directed toward NFI or normal rabbit IgG. HeLa cells were transfected with siRNA directed toward either p300 or GAPDH and were treated with PMA for 10 min. (D and E) Chromatin accessibility by real-time PCR (CHART-PCR) at the c*-fos* promoter. Schematic diagram displays the positions of primers (arrows) used for CHART-PCR analysis. HeLa cells were treated with PMA or TSA for 10 min. Where indicated, cells were pretreated with si-p300 before stimulation. Aliquots of isolated nuclei were incubated with increasing amounts of DNase I (0, 1.0, and 2.0 U), and the relative levels of nuclease protection at the nucleosome positioned at the c*-fos* promoter were measured by real-time PCR. (F) ChIP of the c*-fos* promoter with either an antibody directed toward total histone H3 or acetylated histone H3 from serum-starved HeLa cells (−) or PMA-treated cells (10 min) (+) as indicated. Data in (A), (C), (D), (E), and (F) are presented as means ± SEM (n ≥ 4, 4, 6, 6, and 4, respectively) and are the average of at least two ([A], [C], and [F]) or three ([D] and [E]) independent experiments performed in duplicate.

To establish whether enhanced levels of histone acetylation promote NFI recruitment, we first treated cells with TSA to inhibit HDACs and increase global acetylation levels. This treatment enhanced acetylation levels in the c*-fos* promoter and, importantly, also augmented NFI binding to the c*-fos* promoter (Figure 2A). Furthermore, ChIP analysis on mononucleosomal-associated DNA demonstrated that enhanced histone acetylation occurs on the promoter-proximal nucleosome, which contains the NFI binding site (Figure S2). Next, we depleted the HAT p300 by using siRNA (Figure 2B) and analyzed whether MAP kinase-inducible NFI recruitment was compromised. ChIP analysis demonstrated that inducible NFI recruitment was lost in the absence of p300 (Figure 2C). Upon depletion of p300, NFI recruitment to the promoter after TSA treatment was unaffected, further demonstrating that it is increased acetylation rather than p300 itself that is important for promoting NFI binding (Figure S3).

NFI binding to DNA is not possible in a nucleosomal context when associated with histone octamers (Eisfeld et al., 1997), suggesting that a change in the structure of the nucleosome encompassing the NFI binding site must occur. Histone acetylation can promote changes in nucleosomal structure (reviewed in Workman, 2006); thus, we probed the nuclease sensitivity of the nucleosome in the c*-fos* promoter with chromatin accessibility by real-time PCR (CHART-PCR). Enhanced nuclease sensitivity was apparent upon MAP kinase pathway activation (Figure 2D), indicative of a change in nucleosomal structure. Similar enhancements in nuclease sensitivity could be induced by TSA treatment, whereas p300 depletion reduced the nuclease sensitivity of this region (Figure 2E), demonstrating a role for histone acetylation in this process. Our results therefore predict that NFI binding is a downstream consequence of histone acetylation rather than being important in the generation of the local increase in histone acetylation. This conclusion is further supported by the observation that NFI depletion does not affect histone acetylation levels at the c*-fos* promoter (Figure S4). ChIP analysis with anti-histone H3 antibodies demonstrates that a nucleosome is still present at the promoter, consistent with previous studies that demonstrate its continual presence during the induction process (Figure 2F and Figure S2; Komura and Ono, 2003).

Together, these results suggest a model whereby MAP kinase signaling generates a local increase in histone acetylation. This in turn causes a change in nucleosome structure in the c*-fos* promoter, and these events create a permissive state for NFI recruitment.

### NFI Is Required for MAP Kinase-Mediated Activation of c*-fos*

**Figure 3:**
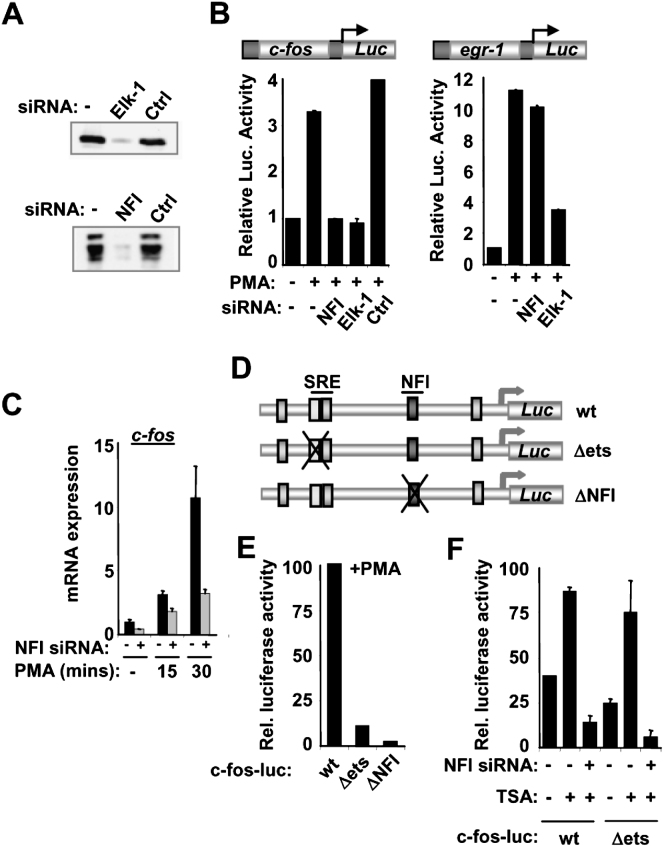
NFI Is Involved in MAP Kinase-Mediated Activation of the c*-fos* Promoter (A) Immunoblot showing the knockdown of Elk-1 protein (top panel) or NFI protein (bottom panel) in HeLa cells 48 hr after transfecting with specifically targeting siRNAs. (B) Luciferase reporter assays with constructs driven by either the c-*fos* promoter (left panel) or the *egr-1* promoter (right panel). HeLa cell lysates were measured for luciferase activity 24 hr after transfection and 6 hr after adding PMA. NFI or Elk-1 was knocked down by siRNA transfection where indicated. (C) Real-time RT-PCR measurement of endogenous c*-fos* mRNA levels after incubation with PMA at the indicated time points and either control (−) or NFI (+) siRNAs. (D) Schematic diagram illustrating luciferase reporter constructs used in (E) and (F). Crosses indicate the mutated elements in each construct. (E and F) Luciferase reporter assays driven by wild-type c*-fos* promoter or promoter regions containing mutations within either the SRE (Δets) or the NFI binding site (ΔNFI). HeLa cell lysates were measured for luciferase activity 24 hr after transfection and 6 hr after adding PMA (E) or TSA (F) as indicated. NFI was knocked down by siRNA where indicated (F). Data in (B), (C), (E), and (F) are presented as means ± SD (n = 3) and are representative of two independent experiments performed in triplicate.

NFI has not been previously linked to c*-fos* regulation in response to MAP kinase pathway activation. We therefore analyzed whether NFI recruitment is important for c*-fos* induction. NFI was depleted by siRNA (Figure 3A), and the activity of a c*-fos* reporter construct was analyzed. Depletion of either Elk-1 or NFI ablated the induction of the c*-fos* promoter (Figure 3B). In contrast, only Elk-1 knockdown had a significant effect on *egr-1* promoter activation, which keeps with the observation that NFI was not recruited to the latter promoter. Similarly, NFI knockdown severely compromised the induction of endogenous c*-fos* expression by either PMA or TNFα (Figure 3C and Figure S5A). Conversely, overexpression of NFI led to an increase in c*-fos* reporter activity (Figure S5B). We also analyzed the importance of the NFI site in the c*-fos* promoter through mutational analysis (Figure 3D). Mutation of either the Elk-1 (ets) or NFI binding sites blocked c*-fos* induction after MAP kinase activation (Figure 3E). Moreover, knockdown of NFI had no effect on the activity of a c*-fos* reporter that lacked the ets site (Figure S5C). Thus, the ets binding site is required for NFI to play a functional role in c*-fos* promoter activation. Importantly, however, in the presence of TSA, although NFI is still required for c*-fos* induction, the ets site is not (Figure 3F). Collectively, these results suggest that the enhanced acetylation caused by TSA is sufficient to overcome the requirement for Elk-1 to activate the promoter through the ets site. However, NFI binding is essential for c*-fos* promoter induction by either MAP kinase activation or by increased acetylation levels by TSA treatment.

### Elk-1-Mediated NFI Recruitment Is Needed for Activation of the Basal Machinery at the c*-fos* Promoter

**Figure 4:**
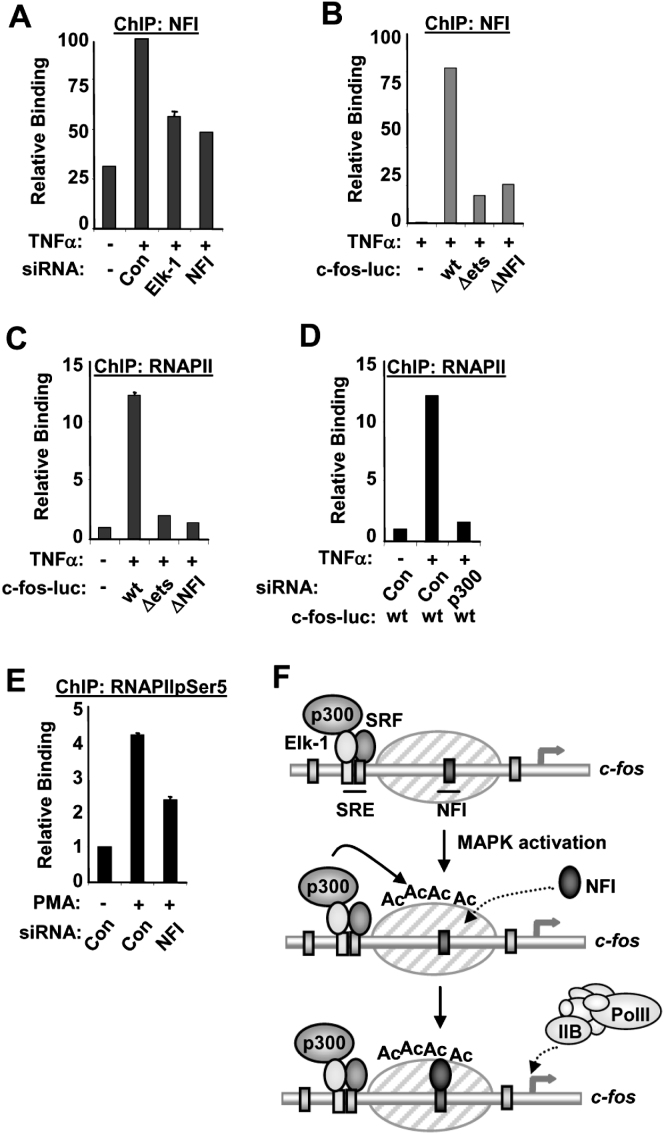
Elk-1 and p300-Mediated MAP Kinase-Induced Recruitment of NFI to the c*-fos* Promoter Represents a Necessary Step in Promoter Activation (A–E) ChIP of endogenous c-*fos* promoter (A and E) or c-*fos*-luciferase reporter DNA (B–D) with either an antibody directed toward NFI (A and B), total RNAPII (C and D), or Ser5-phosphorylated RNAPII (E) from serum-starved HeLa cells or cells treated with PMA or TNFα for 10 min. NFI, Elk-1, or p300 was knocked down by siRNA transfection where indicated. (F) Model of proposed sequential activation of the c-*fos* promoter. MAP kinase pathway activation triggers Elk-1 activation and its associated p300. p300 subsequently acetylates the adjacent nucleosomes, causing a change in nucleosomal structure, NFI recruitment, and the subsequent recruitment and activation of the basal machinery. Data in (A) and (E) are presented as means ± SEM (n ≥ 4) and are the average of at least two independent experiments performed in duplicate. Data in (B), (C), and (D) are presented as means ± SD (n = 3) and are representative of at least two independent experiments performed in triplicate.

Phosphorylation of Elk-1 promotes the allosteric activation of p300 (Li et al., 2003). As p300 knockdown causes a loss of NFI recruitment (Figure 2C), we therefore tested whether Elk-1 binding to the promoter was required for NFI recruitment after MAP kinase pathway activation. Knockdown of Elk-1 reduced the inducible binding of NFI to the c*-fos* promoter (Figure 4A and Figure S6A). Furthermore, mutation of the ets site also blocked NFI recruitment (Figure 4B). These results are consistent with the requirement for the ets motif to enable NFI to function in the regulation of the c*-fos* promoter (Figure S5). c*-fos* activation is accompanied by the recruitment of the basal machinery to the promoter (Ryser et al., 2007). Both the ets and NFI sites are essential for the inducible recruitment of the basal machinery components TFIIB and RNA polymerase II to the c*-fos* promoter after MAP kinase pathway activation (Figure 4C and Figure S6B). Similarly, knockdown of p300 reduces the recruitment of TFIIB and RNA polymerase II (Figure 4D and Figure S6C). Importantly, knockdown of NFI also specifically blocks MAP kinase-induced appearance of activated Ser5-phosphorylated RNA polymerase to the endogenous c*-fos* promoter (Figure 4E).

Thus, consistent with its role in promoting enhanced histone acetylation at the c*-fos* promoter, these results demonstrate that Elk-1 is required for MAP kinase-mediated NFI recruitment and the subsequent engagement of the basal transcription machinery and promoter activation.

## Discussion

High levels of histone acetylation are often associated with active genes, but the molecular mechanisms through which acetylation acts to promote transcription are not well understood (reviewed in Berger, 2002; Workman, 2006). Here we have uncovered a mechanism whereby enhanced histone acetylation activity through one transcription factor (here Elk-1) promotes transcriptional activation through a “HAT-relay switch,” which causes the rapid recruitment of a second transcription factor (here, NFI) (Figure 4F). This secondary recruitment event is essential for the subsequent binding of the basal transcription machinery to the promoter and transcriptional activation. Importantly, this whole process is orchestrated by MAP kinase signaling, which causes enhanced Elk-1 phosphorylation and the resulting change in the local net histone acetylase activity. Thus, these results provide a unique perspective about how MAP kinase signaling leads to gene activation through the inducible indirect recruitment of additional transcription factors to promoters. This mechanism shows some similarities to the regulation of HO transcription in yeast, where an intrinsic signal generated by the cell-cycle machinery activates a phosphatase that in turn triggers Swi5p nuclear import, promoter binding, and histone acetylase recruitment. This then leads to the recruitment of a second transcription factor, SBF, and the subsequent binding of mediator and the general transcriptional machinery (Cosma et al., 1999; Cosma et al., 2001).

Signaling through both the mitogenic (ERK) and stress-activated (p38 and JNK) MAP kinase pathways leads to Elk-1 phosphorylation and subsequent NFI recruitment and target gene activation. However, previous results demonstrated that the amplitude of target gene activation can be influenced by PIASxα, permitting different responses to the ERK and p38 pathway (Yang and Sharrocks, 2006). As PIASxα helps determine the net histone acetylation activity associated with Elk-1, it appears likely that other steps in addition to NFI recruitment might therefore be influenced by changes in acetylation levels. Further studies are, however, required to dissect the subtly different molecular mechanisms that determine these alternative responses.

Although our results on the c*-fos* promoter provide an attractive model, the diversity of mechanisms underlying target gene activation is illustrated by the fact that the well-characterized Elk-1 target gene *egr-1* is activated by MAP kinase signaling in an NFI-independent manner. Thus, different promoters have likely generated specific mechanisms to permit MAP kinase-mediated transactivation to occur. It is possible that similar mechanisms are operative on different genes but that transcription factors other than NFI are inducibly recruited; thus, their identity might not be crucial, but the “transactivating activity” they possess might be more important.

Our results also have more widespread significance, as they provide some parallels with the well-characterized regulation of the MMTV promoter by steroid hormones. Here, the hormonal signals lead to enhanced PR or GR binding, and this in turn causes enhanced NFI recruitment to the MMTV promoter (Di Croce et al., 1999). In this case, however, reciprocal cooperativity in recruitment is also seen with NFI enhancing promoter occupancy by GR (Hebbar and Archer, 2007). Increasing nucleosomal accessibility is again thought to be important in the initial NFI recruitment process, but it is not clear whether histone acetylation has any role in this event. Thus, a common theme emerges in which different signaling pathways can act through their recipient transcription factors (primary effectors) and transmit their signals into transcriptional responses, at least in part, through the recruitment of additional transcription factors (secondary effectors) by altering the local nucleosomal structure. NFI is one such secondary transcription factor in the systems described above, but it is likely that other examples exist.

## Experimental Procedures

### Cell Culture, Luciferase Reporter Assays, and Plasmid Constructs

HeLa cells were maintained in 10% DMEM containing 10% FCS. Where indicated, cells were serum starved for 48 hr and either analyzed immediately or stimulated with TNFα (40 ng/ml), PMA (10 nM) (0–30 min), or TSA (330 nM) (30 min) for an additional time period. When required, cells were pretreated with a mixture of the MAP kinase inhibitors U0126 (10 μM), SB203580 (10 μM), and SP600125 (10 μM) for 30 min.

For luciferase assays, HeLa cells were transfected with Lipofectamine 2000 (Invitrogen) according to the manufacturer's protocol with 400 ng reporter plasmid and 10 ng Renilla luciferase construct (Promega). All results shown are representative of at least three independent experiments analyzed in triplicate. Error bars show standard deviation (n = 3). Reporter constructs used were pFos (−367 to +14)-Luc (pAS2284) and pEgr-luc (kindly provided by Ian Stratford). pFosΔets-Luc (pAS2285) and pFosΔNFI-Luc (pAS2286) were constructed by QuikChange mutagenesis using primer pairs ADS1514/ADS1515 or ADS1672/ADS1673, respectively, with template pAS2284. The NFI expression vector pAS2287 was constructed by inserting a HindIII-XhoI cleaved PCR product (primers ADS1674/ADS1675 and cDNA extracted from HeLa cells) into the same sites in pcDNA3.

### ChIP

ChIP assays in HeLa cells were performed as detailed in the Supplemental Data. All endogenous ChIP results are presented as binding relative to the untreated sample, and error bars are standard error (n ≥ 4). Plasmid-ChIP results are normalized to transfected reporter levels and are representative of at least two independent transfections. Error bars are standard deviation (n = 3).

### CHART-PCR

HeLa cells (60 mm dish) were starved in serum-free DMEM for 48 hr prior to stimulation as indicated in the figures. Cells were harvested in ice-cold PBS. Cell pellets were lysed on ice for 5 min in 350 μl CHART lysis buffer (10 mM Tris [pH 7.4], 10 mM NaCl, 3 mM MgCl_2_, 0.5% NP40, 0.15 mM spermine, and 0.5 mM spermidine). Nuclei were isolated by centrifugation and then washed in 150 μl buffer A (100 mM NaCl, 50 mM Tris [pH 8.0], 3 mM MgCl_2_, 0.15 mM spermine, and 0.5 mM spermidine). After pelleting, nuclei were resuspended in 75 μl buffer A supplemented with 1 mM CaCl_2_ and 12 μl aliquots made to tubes containing various dilutions of DNase I ranging from 0 to 2 U. DNA was digested at 37°C for 3 min, and reaction was stopped by adding 5 mM EDTA, 10 μg Proteinase K, and 1% SDS and incubating at 37°C overnight. DNA was cleaned with QIAquick PCR cleanup columns (QIAGEN) or Chelex (BioRad), and real-time PCR was used to measure the yield of product that is inversely proportional to the accessibility of DNase I to a region of DNA encompassing the positioned nucleosome on the c*-fos* promoter. Primers used cover −205 to −55 are the following: forward, 5′-AGGAACTGCGAAATGCTCAC-3′ (ADS1686), and reverse, 5′-GTAAACGTCACGGGCTCAAC-3′ (ADS1687). A control primer was used to measure a region of c*-fos* at −3395 to −3209: forward, 5′-CAGATCTGCAAATGGCAAAA-3′ (ADS1688), and reverse, 5′-TCTCCTGCCCCACTAACATC-3′ (ADS1689). Results are all presented as level of PCR product relative to the untreated sample, and error bars are standard error (n ≥ 6).

### Quantitative RT-PCR

Total RNA was harvested with an RNeasy kit (QIAGEN). Forty nanograms of RNA was used in a one-step RT-PCR reaction with Quantitect SYBR green reagent (QIAGEN) and the following primers: *18S* forward, 5′-TCAAGAACGAAAGTCGGAGGTT-3′ (ADS4005), and reverse, 5′-GGACATCTAAGGGCATCACAG-3′ (ADS4006); c*-fos* forward, 5′-AGAATCCGAAGGGAAAGGAA-3′ (ADS1690), and reverse, 5′-CTTCTCCTTCAGCAGGTTGG-3′ (ADS1691). c*-fos* levels were routinely normalized to *18S* RNA levels. Results shown are representative of at least two independent experiments measured in at least triplicate. Error bars show standard deviation (n = 3).

### siRNA

Where indicated, HeLa cells were transfected with 50 nM siRNA targeting p300, Elk-1, or GAPDH (Dharmacon), NFI (Santa Cruz), or nontargeting control siRNA (Santa Cruz), using oligofectamine (Invitrogen). Cells were left for 48 hr and then treated with TSA or MAP kinase pathway inducers where required.

### Western Blot Analysis

Western blotting was carried out with Supersignal West Dura Extended Duration Substrate (Pierce) and primary antibodies anti-Elk-1 (Santa Cruz), anti-phospho-JNK, anti-phospho-ERK, anti-phospho-p38 (Cell Signaling), anti-GAPDH (Abcam), anti-p300 (Santa Cruz), or anti-NFI (Santa Cruz). Data were visualized with a Bio-Rad Fluor-S MultiImager and Quantity One software (Bio-Rad).

## Supplemental Data

Document S1. Supplemental Experimental Procedures and Six Figures
