# Supplementary material for: MAP Kinase-Mediated c-fos Regulation Relies on a Histone Acetylation Relay Switch
Source: Mol Cell. 2008 Mar 28;29(6):780–5. doi: 10.1016/j.molcel.2008.01.019 (PMC3574235; doi:10.1016/j.molcel.2008.01.019)
Supplement: Document S1. Supplemental Experimental Procedures and Six Figures [file mmc1.pdf]

Molecular Cell, Volume *29*

Supplemental Data

MAP Kinase-Mediated *c-fos* Regulation Relies on a Histone Acetylation Relay Switch

Amanda O'Donnell, Shen-Hsi Yang, and Andrew D. Sharrocks

Supplemental Experimental Procedures

Chromatin Immunoprecipitation analysis

HeLa cells were treated with 1% formaldehyde for 10 min at room temperature before quenching with 0.125 M glycine for 5 min. For standard ChIP, cells were harvested in ice-cold PBS with complete protease inhibitors (Roche) and washed sequentially with Buffer I (10 mM HEPES pH 6.5, 0.5 mM EGTA, 10 mM EDTA, 0.25% Triton X-100) and Buffer II (10 mM HEPES pH 6.5, 0.5 mM EGTA, 1 mM EDTA, 200 mM NaCl) then resuspended in SDS lysis buffer (50 mM Tris pH 8.1, 10 mM EDTA, 1% SDS). Lysates were sonicated on ice to yield 200-600 bp DNA fragments for endogenous ChIP and 1-2 kb for plasmid-ChIP.

To produce mononucleosomal preparations for ChIP analysis, cells were crosslinked as usual and were harvested in PBS. Following centrifugation at 700g, pellets were then resuspended in 10 volume of Sucrose buffer A (0.32 mM Sucrose, 15 mM HEPES, 60 mM KCl, 2 mM EDTA, 0.5 mM EGTA, 0.5% BSA, 0.5 mM spermidine, 0.15 mM spermine, 0.5 mM DTT and protease inhibitor cocktail). Cells were disrupted with a Dounce homogeniser and the nuclear suspension was layered over an equal volume of Sucrose buffer B (0.32 mM Sucrose, 15 mM HEPES, 60 mM KCl, 2 mM EDTA, 0.5 mM EGTA, 0.5 mM spermidine, 0.15 mM spermine, 0.5 mM DTT and protease inhibitor

cocktail) and spun down 3000 rpm for 15 mins. The purified nuclei were collected and resuspended in 1 pellet volume of buffer NUC (15 mM HEPES, 60 mM KCl, 15 mM NaCl, 0.34 mM sucrose, 0.15 mM β-mercaptoethanol, 0.5 mM spermidine, 0.15 mM spermine and protease cocktail. $CaCl_2$ was added to the nuclei to final concentration of 3 mM and immediately 100 U of micrococcal nuclease was added. Reactions were performed at 37°C for 30 mins and stopped by adding an equal volume of 2x Sonication buffer (90 mM HEPES, 220 mM NaCl, 10 mM EDTA, 2% Triton X-100, 0.2% Na-deoxycholate, 0.2% SDS, 0.5 mM PMSF and protease inhibitor cocktail). The sample was briefly sonicated and spun down at 14,000 rpm for 15 mins.

One quarter of a 10cm dish was used per endogenous ChIP and one quarter of a well in a 6-well dish used per plasmid ChIP, diluted 1/10 in IP Dilution buffer (0.01% SDS, 1.1% TRITON-X-100, 1.2 mM EDTA, 16.7 mM TrisHCl pH 8.1, 167 mM NaCl) and incubated overnight at 4°C with either 1 μg anti-Elk-1 (Santa Cruz), anti-acetylated histone H4 (Upstate), anti-NFI (Santa Cruz), anti-acetylated histone H3 (phosphor Ser10-acetyl Lys14) (Upstate), anti-Histone H3 (Upstate), anti-TFIIB (Santa Cruz), anti-RNAPII (Santa Cruz), anti-RNAPII phosphoSer-5 (Covance) or 1μg non-specific IgG (Upstate). Immunocomplexes were precipitated by incubation for 60 minutes with protein A-conjugated (or anti-mouse IgM with anti-RNAPII phosphoSer-5 antibody) magnetic beads (Dynal) that had been pre-blocked by incubation with 2μg salmon sperm DNA. Immunoprecipitates were washed sequentially with TSEI (20 mM, Tris pH 8.1, 2 mM EDTA, 150 mM NaCl, 1% Triton, 0.1% SDS,), TSEII (20 mM Tris pH 8.1, 2 mM EDTA, 500 mM NaCl, 1% Triton, 0.1% SDS), Buffer III (10 mM Tris pH 8.1, 0.25 M LiCl, 1mM EDTA, 1% NP40, 1% DOC) and TE before eluting in 1%SDS/ 0.1M

$NaHCO_3$. Cross-links were reversed by heating to 65°C overnight, then treating with proteinase K for 1 h at 45°C. Chromatin was cleaned using QiaQuick PCR cleanup columns (Qiagen). PCR was performed using specific primers to human *c-fos* promoter (-353 to -189 relative to transcription start site), forward- GAGCAGTTCCCGTCAATCC (ADS1676), reverse- GCATTTCGCAGTTCCTGTCT (ADS1677); *egr-1* promoter (-606 to -381), forward- GCTTCCCCAGCCTAGTTCAC (ADS1644), reverse-TGCCCAAATAAGGGTTGTTC (ADS1645); *c-fos* start site (-71 to +174), forward-GAGCCCGTGACGTTTACACT (ADS1680), reverse-TTGAAGCCCGAGAACATCAT (ADS1681); pFos-Luc plasmid-ChIP (*luc* coding region), forward- CCAGGGATTTCAGTCGATGT (ADS1682), reverse-AATCTGACGCAGGCAGTTCT (ADS1683). For amplification of the *c-fos* promoter-proximal nucleosome on mononucleosome-associated DNA preparations, the primer pair ADS1172 (Primer4F); GGGGGGAGCCATCCCCGAAA and ADS1173 (Primer4R) GCTTCTGCGGCCGCCCGGCT (-110 to -240) was used. Quantitative PCR was performed in at least duplicate, from at least two independent experiments, using Quantitect SYBR green PCR reagent (Qiagen) and a Rotorgene 3000 machine (Corbett Research). Results were analysed with Rotorgene 6.0 software (Corbett Research) relative to input using the standard curve method. Specific endogenous ChIP enrichments were all at least 3-fold over normal IgG ChIP, and at least 3-fold more than ChIP of an intronic region of the *SRF* gene (+5043 to +5224), forward-GCCACAGGGCAGTAGATGTT (ADS1684), reverse-TCAGGCCCAAGTATCCACTC (ADS1685).

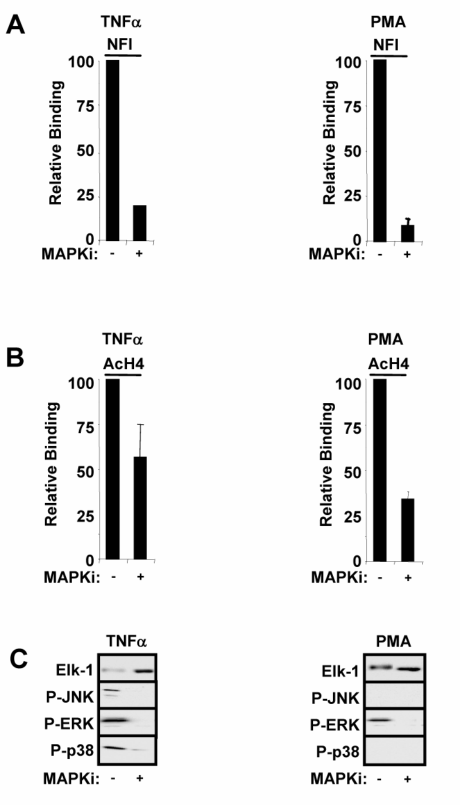

Figure S1. NFI is recruited to the *c-fos* promoter in response to MAP kinase activation. (*A-B*) Chromatin immunoprecipitation of the *c-fos* promoter with antibody towards either NFI (*A*) or acetylated histone H4 (*B*). Amplified DNA was detected using primer pair ADS1676/ADS1677. No enrichment of chromatin by NFI was found at non-promoter regions (data not shown). (*C*) Immunoblots of the indicated activated components of the MAP kinase pathway. Left panels show activation by TNFα and right panels by PMA. HeLa cells were starved in serum-free DMEM for 48h before treating with TNFα or PMA for 10 minutes. Where indicated, cells were pre-treated with MAP kinase inhibitors (MAPKi) U0126 (10μM), SB203580 (10μM) and SP600125 (10μM) (*A*) or just U0126 (10μM) (*B*) for 30 minutes. Following treatment with TNFα, the activated phosphorylated forms of JNK, ERK and p38 are detected (P-) whereas PMA only activated ERK. A mixture of MAP kinase inhibitors reduces this activation, and in the

case of PMA stimulation, U0126 is sufficient (data not shown). This activation/ inactivation cycle is accompanied by similar changes in Elk-1 phosphorylation levels (as revealed by changes in phosphorylation-induced mobility shift). Treatment with the MAP kinase inhibitors reduces histone acetylation (*B*) and NFI recruitment (*A*) to the *c-fos* promoter. Data in parts A and B are presented as SEM (n≥4) and are the average of at least two independent experiments performed in duplicate.

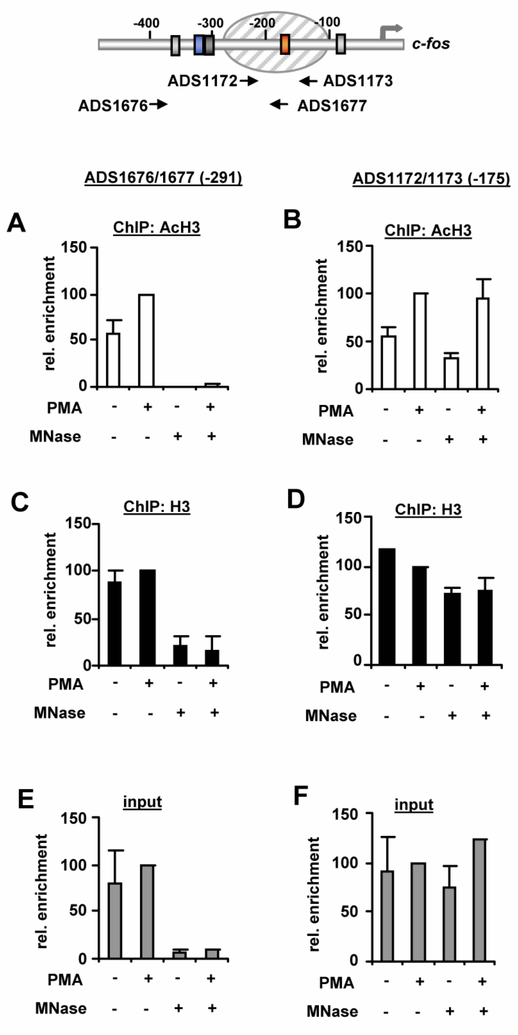

Figure S2. PMA-induced histone acetylation occurs on the promoter-proximal nucleosome. The schematic at the top of the figure illustrates the locations of the primers used for ChIP relative to the transcriptional start site and other elements of the promoter (see Fig. 1 for details of other elements). The centre of the regions covered by the primer pairs are indicated. ChIP assays were performed in Hela cells that had previously been treated with PMA where indicated. Both total histone H3 levels (C and D) and the levels of acetylated histone H3 (A and B) binding to the *c-fos* promoter were analysed. Levels of input DNA are also shown (E and F). Two distinct primer pairs which spanned different overlapping regions of the promoter (top panel). Binding to the promoter-

proximal nucleosome were analysed using the primer pair (ADS1172/ADS1173) which are centred around position -175 relative to the transcriptional start site. Where indicated, nucleosomal DNA was subjected to micrococcal nuclease (MNase) digestion prior to ChIP analysis to generate mononucleosomal chromatin. Data in parts A and B are presented as SEM (n=6) and are the average of three independent experiments performed in duplicate.

Panels A, C and E clearly demonstrate the quality of the micrococcal nuclease digestion as the signals are lost when primers span the edge of the nucleosome. Importantly, increases in histone acetylation are seen with both primer pairs used on mononucleosomal DNA demonstrating that the PMA-inducible acetylation event is occurring on the promoter-proximal nucleosome that encompasses the NFI binding site.

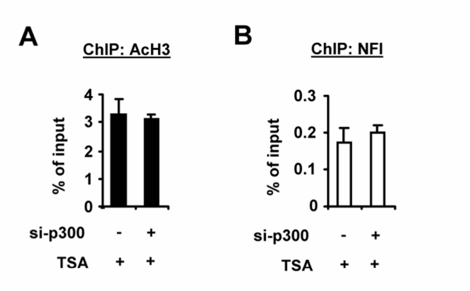

Figure S3. p300 is not required for TSA-mediated NFI binding. ChIP assays were performed in Hela cells that had previously been treated with TSA in the presence and absence of siRNA duplexes targeted to p300. Both NFI levels and the levels of acetylated histone H3 binding to the promoter-proximal nucleosome were analysed using the primer pair (ADS1172/ADS1173) and mononucleosomal chromatin. Data in parts A and B are presented as means ±SEM (n=2) and are representative of two independent experiments performed in duplicate. No differences in NFI binding or histone acetylation were observed upon knockdown of p300, indicating that TSA was able to enhance acteylation levels in a p300-independent manner. This further substantiates the conclusion that histone acetylation is sufficient for promoting NFI binding to the *c-fos* promoter.

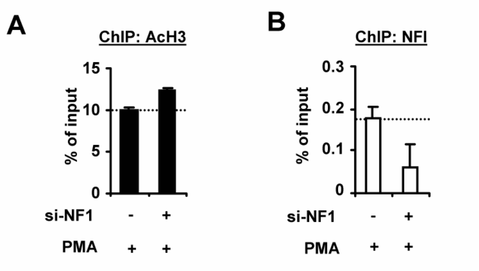

Figure S4. Depletion of NFI does not affect histone acetylation levels. ChIP assays were performed in Hela cells that had previously been treated with PMA in the presence and absence of siRNA duplexes targeted to NFI. Both NFI levels and the levels of acetylated histone H3 binding to the promoter-proximal nucleosome were analysed using the primer pair (ADS1172/ADS1173) and mononucleosomal chromatin. Data in parts A and B are presented as means ±SEM (n=2) and are representative of two independent experiments performed in duplicate.

Upon depletion of NFI, acetylation of the promoter-proximal nucleosome is still detected, demonstrating that NFI binding is an event downstream rather than upstream from histone acetylation at this locus. Moreover, this experiment confirms that we can detect NFI association with the promoter-proximal nucleosome.

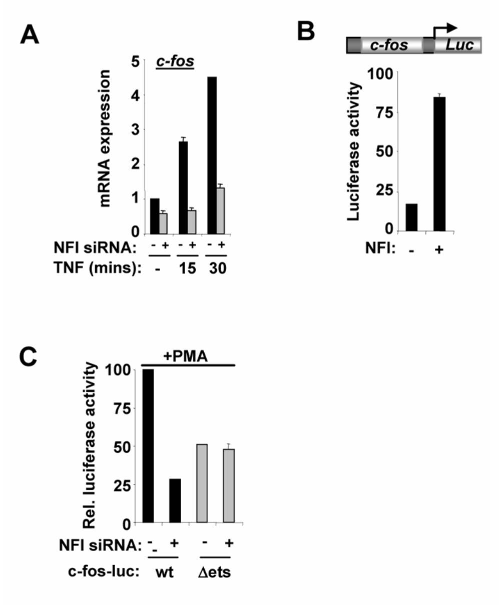

Figure S5. NFI is involved in MAP kinase-mediated activation of the *c-fos* promoter. (*A*) Real-time RT-PCR measurement of endogenous *c-fos* mRNA levels in Hela cells after treatment with TNFα at the indicated time-points. NFI was knocked down by siRNA transfection where indicated (+). (*B,C*) Luciferase reporter assays driven by the wild-type (wt) or a mutant (Δets) *c-fos* promoter. (*B*) NFI expression vector (50 ng) was co-transfected with the reporter where indicated (+). (*C*) HeLa cell lysates were measured for luciferase activity 24h after transfection and 6h after adding PMA. NFI was knocked down by siRNA transfection where indicated (+).Data in parts A, B and C are presented as means ±SD (n=3) and are representative of two independent experiments performed in triplicate.

These data demonstrate that NFI can activate the *c-fos* promoter when overexpressed (*B*). Furthermore, depletion of NFI reduces the ability of PMA to activate the wild-type *c-fos* promoter, but is ineffective once the Elk-1 binding site is removed in the Δets construct (*C*). The increase in basal activity of the *c-fos* promoter upon mutation of the ets motif most likely reflects the loss of binding of Elk-1 which represses the *c-fos* promoter under

basal conditions (Yang et al., 2003a). As observed with PMA, NFI depletion diminishes the activation of *c-fos* in response to TNFα treatment (*A*).

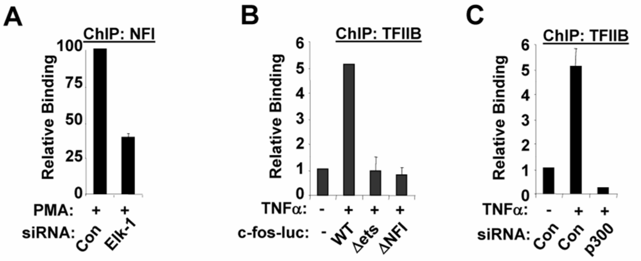

Figure S6. Elk-1 and p300 mediated MAP kinase-induced recruitment of NFI to the *c-fos* promoter represents a necessary step in promoter activation. Chromatin immunoprecipitation of endogenous *c-fos* promoter (*A*) or *c-fos*-luciferase reporter DNA *(B, C*) using either an antibody directed towards NFI (*A*) or TFIIB (*B, C*) from serum starved HeLa cells (-) or Hela cells treated with PMA or TNFα for 10 minutes (+). Elk-1 or p300 were knocked down by siRNA where indicated. Data in part A are presented as means ±SEM (n=4) and the average of two independent experiments performed in duplicate. Data in parts B and C are presented as means ±SD (n=3) and are representative of two independent experiments performed in triplicate.

As observed with TNFα, Elk-1 is needed for NFI recruitment following PMA treatment (*A*). The TNFα-induced recruitment of TFIIB to the promoter is dependent on the binding motifs for Elk-1 and NFI (*B*) and adequate p300 levels in the cell (*C*).
